# Revisiting MSC heterogeneity: significance of the CD73^+^CD39^+^CD146^+^ perivascular subset in bone marrow

**DOI:** 10.3389/fbioe.2026.1810362

**Published:** 2026-04-15

**Authors:** Ramya Lakshmi Rajendran, Atharva A. Mahajan, Byeong-Cheol Ahn, Prakash Gangadaran

**Affiliations:** 1 BK21 FOUR KNU Convergence Educational Program of Biomedical Sciences for Creative Future Talents, Department of Biomedical Sciences, School of Medicine, Kyungpook National University, Daegu, Republic of Korea; 2 Department of Nuclear Medicine, School of Medicine, Kyungpook National University, Daegu, Republic of Korea; 3 Cardiovascular Research Institute, Kyungpook National University, Daegu, Republic of Korea; 4 Cancer Research Institute, Advanced Centre for Treatment Research and Education in Cancer, Navi Mumbai, Maharashtra, India; 5 Department of Nuclear Medicine, Kyungpook National University Hospital, Daegu, Republic of Korea

**Keywords:** bone marrow niche, CD73^+^CD39^+^CD146^+^ subset, mesenchymal stromal cells (MSC), perivascular MSCs, stromal heterogeneity

## Outline

The recent article by Martin and Gullo (2025) identifies and isolates a CD73^+^CD39^+^CD146^+^MSC subset from bone marrow, demonstrating its self-renewal capacity, multipotency, perivascular localization, and adenosine production. This rare subset is also detectable in mobilized blood, suggesting potential applications for HSC co-culture and enhanced transplant readiness. This opinion explores the work of Martin and Gullo (2025), emphasizing the significance of the CD73^+^CD39^+^CD146^+^ subset while addressing the study’s limitations.

### Significance of the triple-positive MSC subset

The isolation of the CD73^+^CD39^+^CD146^+^ population is significant because it provides a precise functional annotation of the perivascular niche, moving beyond the heterogeneous definitions that often complicate MSC research (Wiley, 2026). While CD146 (also known as the melanoma cell adhesion molecule, MCAM) is a well-established marker of pericytes and clonogenic stromal cells that regulate hematopoiesis via factors such as CXCL12 and Angiopoietin-1, the authors demonstrated that including CD39 (ecto-NTPDase) and CD73 (ecto-5′-nucleotidase) defines a population with superior metabolic capacity. This specific phenotype suggests that these cells are not merely structural supports but actively regulate the local microenvironment by converting extracellular ATP to adenosine. The functional relevance of this profile is underscored by the finding that the sorted subset produced significantly higher concentrations of adenosine (21.6 pmol/μL) compared to the unsorted bone marrow population (16.4 pmol/μL). This aligns with previous findings in synovial tissue, where this profile indicated greater osteo-chondrogenic potency, a trait now confirmed in the bone marrow context, where the subset exhibited osteogenic potential exceeding that of osteoblasts (Petinati et al., 2020).

### Methodological strengths

The study (Martin and Gullo, 2025) employs a robust methodological approach to validate this subset beyond the mere expression of surface markers.

- High-Purity Sorting: The sorting strategy, utilizing doublet exclusion and viability gating, achieved a high purity (>90%) of the co-expressing population, ensuring that downstream functional assays were specific to this subset.
- *In-situ* Mapping: Immunofluorescence on bone marrow core slides confirmed that the CD73^+^CD39^+^CD146^+^ phenotype is a genuine physiological resident of the perivascular niche, rather than an artifact resulting from *ex vivo* culture expansion.
- Functional Validation: The study moved beyond immunophenotyping to demonstrate “stemness” via standard ISCT criteria (plastic adherence, clonogenicity, and trilineage differentiation) and functional enzymatic activity via adenosine fluorometric assays.

### Novel insights offered by the study

Two key insights distinguish this work (Martin and Gullo, 2025) from previous bulk MSC studies, providing a more detailed understanding of stromal function and trafficking.

1. Metabolic Superiority: The study extends beyond surface marker profiling to establish a functional metabolic hierarchy within the bone marrow stroma. The isolated triple-positive subset produced significantly higher levels of adenosine (21.6 pmol/μL) compared to the unsorted bone marrow population (16.4 pmol/μL). Since the sequential action of CD39 and CD73 converts pro-inflammatory extracellular ATP into immunosuppressive adenosine, this enhanced enzymatic activity suggests that metabolic regulation of immune signaling is a defining characteristic of the primitive stromal niche (Reply, 2017). This finding implies that this specific subset functions as a “metabolic gatekeeper,” likely maintaining the low-inflammatory environment necessary for HSC quiescence.
2. Mobilization Dynamics: The detection of this subset in mobilized blood is a novel finding that challenges the traditional view of perivascular MSCs as strictly tissue-resident. Although the overall frequency in the apheresis product was notably low (<1%), the study revealed a striking phenotypic divergence based on the mobilization regimen. In samples mobilized with G-CSF alone, approximately 80% of the circulating CD73^+^CD39^+^ cells retained CD146 expression. However, dual mobilization with G-CSF and Plerixafor resulted in a distinct shift, with only about 25.1% of the CD73^+^CD39^+^ cells retaining CD146 expression. This unexpected reduction suggests that Plerixafor—a CXCR4 antagonist—may differentially influence the adhesion or release mechanisms of the CD146^+^ pericyte fraction compared to the CD146^-^ stromal population (Konopleva et al., 2015). The observed reduction in CD146 expression following dual mobilization with G-CSF and Plerixafor should be interpreted cautiously. While CXCR4 antagonism may influence perivascular cell detachment, alternative explanations must be considered (Fricker, 2013; Pelus and Farag, 2011; Alipourfard et al., 2026). These include selective mobilization of distinct stromal subsets, potential shedding, or downregulation of CD146 during mobilization, and phenotypic plasticity of stromal cells upon entry into circulation. Further mechanistic studies are required to delineate whether these changes reflect true biological reprogramming or transient phenotypic modulation. A comparative overview of the phenotypic distribution and functional characteristics of this subset across bone marrow and mobilized blood sources is summarized in Table 1.

**TABLE 1:** Comparative profile of the CD73^+^CD39^+^CD146^+^ MSC subset by source.

Parameter	Bone marrow (BM)	Single-mobilized PBSCs (G-CSF)	Dual-mobilized PBSCs (G-CSF + plerixafor)	Non-mobilized blood (MNCs)
Mobilization strategy	—	G-CSF (filgrastim)	G-CSF + plerixafor	None
Subset detection	High (in culture-expanded BM)	Present	Present	Not detected
Frequency	80%–96% (culture-expanded; passage 2–3)	<1% (CD45^−^ fraction)	<1% (CD45^−^ fraction)	0%
CD146 expression within CD73^+^CD39^+^ cells	∼100%	∼80%	∼25%	N/A
Localization	Perivascular niche (bone marrow)	Circulating	Circulating	N/A
Adenosine production	High (21.6 pmol/μL vs. 16.4 in bulk BM)	Not assessed	Not assessed	N/A
Differentiation capacity	Tri-lineage (osteogenic, adipogenic, chondrogenic)	Not assessed	Not assessed	N/A

BM, bone marrow; PBSCs, peripheral blood stem cells; MNCs, mononuclear cells; G-CSF, granulocyte colony-stimulating factor; CD, cluster of differentiation; CD45^−^, non-hematopoietic cell fraction; CD73, ecto-5′-nucleotidase; CD39, ectonucleoside triphosphate diphosphohydrolase-1 (ENTPD1); CD146, melanoma cell adhesion molecule (MCAM); N/A, not applicable.

### The primary limitation of this study

The CD73^+^CD39^+^CD146^+^ subset should be interpreted within the established framework of bone marrow stromal heterogeneity (Martin and Gullo, 2025). Previous studies have identified LepR^+^ stromal cells, Nestin^+^ MSCs, NG2^+^ pericytes, and CXCL12-abundant reticular (CAR) cells as key regulators of hematopoietic stem cell (HSC) maintenance (Crane et al., 2017; Omatsu, 2023; Zhou et al., 2017). Notably, CD146^+^ perivascular cells substantially overlap with NG2^+^ pericytes and LepR^+^ stromal populations (Asada et al., 2017), suggesting that the triple-positive subset may represent a metabolically specialized fraction within these compartments rather than a completely distinct lineage. The defining feature of this subset may therefore lie in its enhanced purinergic metabolism, rather than its anatomical exclusivity.

### Implications for hematopoietic stem cell biology

These findings have profound implications for HSC engineering. Since pericytes secrete CXCL12 and Angiopoietin-1 factors critical for HSC retention and quiescence, this CD146^+^ subset is likely the physical anchor of the HSC niche (Wang et al., 2019). The demonstrated high capacity for adenosine production further suggests that this subset creates an immunosuppressive “shield,” protecting HSCs from inflammatory stress. The author’s proposal to use this subset in co-culture systems offers a promising strategy to mimic the *in vivo* niche, potentially enhancing the expansion of primitive HSCs for transplantation.

### Opportunities for future work

While the study (Martin and Gullo, 2025) lays a strong foundation, several areas warrant further investigation.

1. Yield and Scalability: The frequency of this subset in mobilized leukopaks was very low (<1%) (Gao et al., 2023). Future studies should determine whether clinical-grade expansion protocols can produce sufficient numbers of these cells without inducing senescence or phenotypic drift.
2. Mobilization Mechanism: The data indicate that Plerixafor (a CXCR4 antagonist) does not significantly increase the yield of this subset compared to G-CSF alone. Investigating why these pericytes are resistant to CXCR4 antagonism could reveal unique adhesion mechanisms that anchor them to the vasculature (Beck et al., 2014).
3. Loss of CD146: The significant decrease in CD146 expression in dual-mobilized samples (from approximately 80%–25%) warrants a mechanistic explanation. Does Plerixafor treatment induce shedding of CD146 (Todorova et al., 2023), or does it selectively mobilize a CD146-negative subpopulation?
4. The concept of utilizing this subset as a “nurse cell: A few previous studies employing MSC feeder layers, biomimetic scaffolds, and niche-inspired cytokine cocktails have demonstrated partial success in preserving HSC stemness *ex vivo* (Bourgine et al., 2018; Xiao et al., 2022; Bessy et al., 2021). In this context, the CD73^+^CD39^+^CD146^+^ subset may offer an advantage by integrating structural support with metabolic regulation via adenosine signaling, thereby more closely recapitulating the native niche environment.

## Conclusion

In conclusion, the study by Martin and Gullo (2025) provides a robust immunophenotypic and functional framework for defining the CD73^+^CD39^+^CD146^+^ subset as a critical component of the perivascular niche. By demonstrating that this subset combines superior adenosine-generating capacity with retained “stemness” in both bone marrow and mobilized blood, the authors have identified a potent candidate for “nurse cell” applications in hematopoietic engineering. Although challenges such as low yield in apheresis products and phenotypic instability under dual mobilization remain to be addressed, the metabolic and structural uniqueness of this triple-positive population suggests that targeting it rather than bulk MSCs represents a paradigm shift toward precision stromal therapy for enhanced HSC expansion and transplantation.
